# Effects of Circular DNA Length on Transfection Efficiency by Electroporation into HeLa Cells

**DOI:** 10.1371/journal.pone.0167537

**Published:** 2016-12-05

**Authors:** Benjamin D. Hornstein, Dany Roman, Lirio M. Arévalo-Soliz, Melinda A. Engevik, Lynn Zechiedrich

**Affiliations:** 1 Department of Molecular Virology and Microbiology, Baylor College of Medicine, Houston, TX, United States of America; 2 Post-Baccaleureate Research Education Program, Baylor College of Medicine, Houston, TX, United States of America; 3 Verna and Marrs Mclean Department of Biochemistry and Molecular Biology, Baylor College of Medicine, Houston, TX, United States of America; 4 Department of Pharmacology, Baylor College of Medicine, Houston, TX, United States of America; 5 Department of Pathology, Texas Children’s Hospital, Houston, TX, United States of America; Universidad de Castilla-La Mancha, SPAIN

## Abstract

The ability to produce extremely small and circular supercoiled vectors has opened new territory for improving non-viral gene therapy vectors. In this work, we compared transfection of supercoiled DNA vectors ranging from 383 to 4,548 bp, each encoding shRNA against GFP under control of the H1 promoter. We assessed knockdown of GFP by electroporation into HeLa cells. All of our vectors entered cells in comparable numbers when electroporated with equal moles of DNA. Despite similar cell entry, we found length-dependent differences in how efficiently the vectors knocked down GFP. As vector length increased up to 1,869 bp, GFP knockdown efficiency per mole of transfected DNA increased. From 1,869 to 4,257 bp, GFP knockdown efficiency per mole was steady, then decreased with increasing vector length. In comparing GFP knockdown with equal masses of vectors, we found that the shorter vectors transfect more efficiently per nanogram of DNA transfected. Our results rule out cell entry and DNA mass as determining factors for gene knockdown efficiency via electroporation. The length-dependent effects we have uncovered are likely explained by differences in nuclear translocation or transcription. These data add an important step towards clinical applications of non-viral vector delivery.

## Introduction

Gene therapy, or the use of nucleic acids to regulate, replace, or repair genes to prevent or treat human disease, is an emerging technology to treat or prevent disease [1]. In the past few decades, hundreds of gene therapy candidate genes have been uncovered [2,3], yet very few of these have turned into target therapies because of the rate-limiting step of gene therapy–the delivery of the nucleic acid. Synthetic short interfering RNAs (siRNAs), viral vectors, plasmid vectors, and minimized DNA vectors (minicircles/minivectors) have all been utilized as gene therapy delivery tools. Each tool has advantages and disadvantages, and optimizing each for human use is of high priority [1].

Viral vectors are highly efficient at gene delivery, but have potential risks [4]. Non-viral vectors do not have many of the risks associated with viral vectors, but are generally less efficient at delivering genes. Engineering of plasmids can improve expression, persistence, and immunogenicity [5,6]. Non-viral DNA vectors that have had the bacterial origin of replication and antibiotic resistance-encoding genes removed are known as minicircles or minivectors. Minivectors can be smaller and more negatively supercoiled than minicircles, making them more compact and more resistant to shear forces [7,8].

Reducing non-viral DNA vector length has been demonstrated in previous studies to improve transfection efficiency and persistence in cells [9–11], and to increase survival to the shearing forces of nebulization or sonication [8], Most previous work on transfection efficiency with minicircles, however, failed to differentiate the effects of removing bacterial sequences from the effects of reducing vector length. Investigations of DNA vector length did not include vectors shorter than 2,900 bp [11]; studies that included vectors shorter than 2,900 bp did not investigate vector length as an independent variable [12,13]. Instead, most work compared minicircles to their parent plasmids only, and not among vectors of different lengths [12,13].

When assessing how vector length affects transfection, the mode of vector delivery is an important consideration. Many transfection studies use cationic lipid delivery vehicles, such as Lipofectamine. Lipofectamine forms liposomes of similar sizes regardless of the size of the DNA vector [11]; transfecting equal moles of different vectors with Lipofectamine requires normalizing the total DNA mass with additional, non-coding plasmid DNA to keep the total charge identical [7,9,10,12]. Successful liposomal transfection requires endosomal escape [14], which may be affected by vector length, or the vehicle itself. Although nonviral vectors transfected via electroporation are also subjected to endosomal trafficking [15,16], they are not affected by the amount of delivery vehicle present. Because of the confounding issues inherent to delivery vehicles, here we used electroporation to assess transfection of DNA vectors of eight different lengths, ranging from 383 to 4,548 bp, into HeLa cells.

“Transfection” is considered to be either the process of vector entry through the cell membrane [17] or the resulting expression from the transgene [18]. In this study, we distinguished these two aspects of transfection. The surprising results we present here provide a greater understanding of DNA transfection by electroporation and are an important step towards optimization of non-viral gene therapy for clinical applications.

## Results

### Experimental rationale

DNA vectors of different lengths have different molecular weights. An identical mass (*e*.*g*., ng) of different sized vectors will have differing numbers of molecules (*e*.*g*., fmol), and *vice versa*. Therefore, we analyzed transfection efficiency via electroporation both with respect to mass and with respect to moles of DNA transfected to determine how vector length affects transfection.

To electroporate our vectors into cells, we used the Neon transfection system. This system applies an electric field within a pipette tip customized with a gold wire-type electrode [19]. The optimal settings for transfecting plasmids into HeLa cells using the Neon transfection system are two pulses of 1,050 V, 30 ms in length. This voltage corresponds to a field strength of 420 V/cm (this value may not accurately translate to an equivalent field strength of a traditional, cuvette-based electroporation system). We found that plasmids and minivectors transfected similarly when the voltage was varied between 850 V and 1,250 V, the pulse width was varied between 20 and 40 ms, and either one and two pulses were tested (data not shown). Concurrently, measuring toxicity using NucBlue cell stain, we observed less than 10% cell death with these parameters; voltage higher than 1,050 V or pulse widths greater than 30 ms (Neon recommended settings for HeLa cells) drastically reduced cell density, suggesting high cell toxicity when transfecting either minivectors or plasmids. We therefore used the manufacturer’s optimized plasmid settings to electroporate minivectors into HeLa cells.

**Fig 1:**
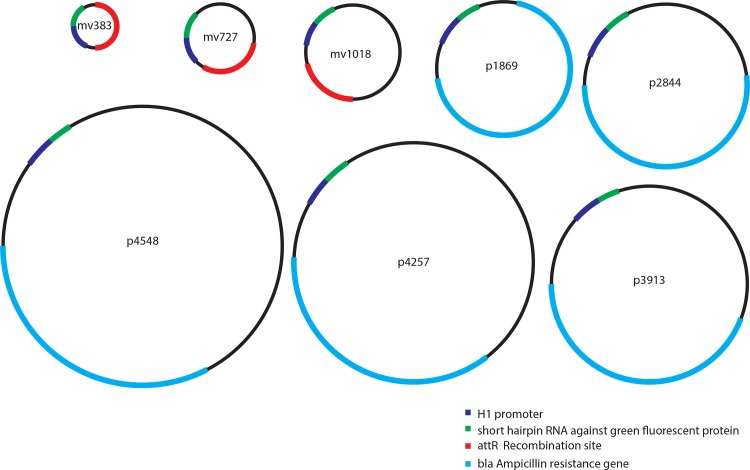
Schematic of DNA vectors tested. Vectors are drawn to relative scale. p = plasmid; mv = minivector. The numbers denote length in base pairs.

Fluorescent reporter genes such as those encoding green fluorescent protein (GFP) or luciferase, are too long to allow us to assess our shortest vectors. Instead, we encoded an shRNA under control of the H1 promoter on DNA vectors and utilized shRNA-mediated GFP knockdown in HeLa cells stably expressing a short-lived GFP (hereafter refer to as "HeLa-GFP"). This GFP has a half-life of only two hours, making knockdown highly quantifiable [20]. We designed, cloned, and purified 383, 727, 1,018, 1,869, 2,844, 3,913, 4,257, or 4,548 bp DNA vectors, each with identical H1 promoter-GFP shRNA cassettes (Fig 1).

### Effect of DNA vector length on cell entry

**Fig 2:**
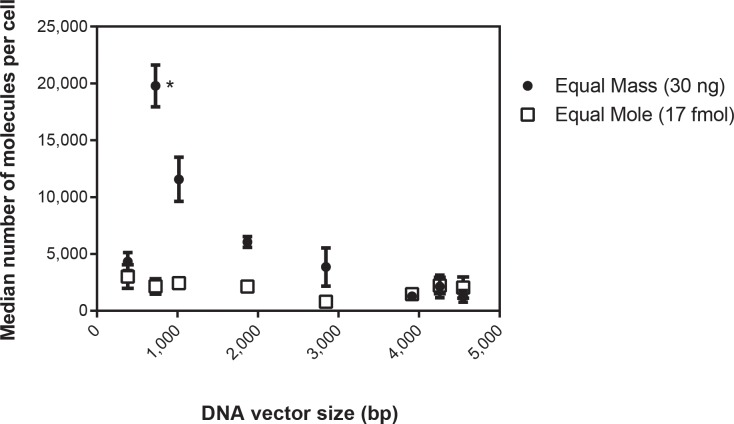
Effect of DNA vector length on cell entry. Each of the DNA vectors was labeled with Cy3 using LabelIT and transfected into 90,000 HeLa-GFP cells. Cells were transfected using electroporation with 30 ng of each of the eight DNA vectors and the median Cy3 fluorescence was determined using flow cytometry (circles). In a separate experiment, 90,000 HeLa-GFP cells were transfected with 17 fmol of each of the eight DNA vectors (equivalent to 30 ng of the p2844) (squares). Median Cy3 fluorescence was compared to a standard curve of R-PE beads to determine the number of fluorophores, which, when combined with the number of Cy3 fluorophores per DNA molecule for each vector, yielded the number of molecules per cell. *For transfections at equal mass, results for mv727 were statistically significantly higher (by one-way ANOVA) than the results for all the vectors except mv1018. No other pairwise comparisons were statistically significant.

For vectors to cause GFP knockdown, they must penetrate the cellular and nuclear membranes and then be recognized and transcribed by an RNA polymerase. Vector length could affect any of these steps. To first assess cell entry, vectors were labeled with Cy3-LabelIT and electroporated into HeLa-GFP cells either with the same DNA mass (30 ng) or with the same moles (17 fmol). These vector concentrations were chosen because they knocked down about half of the GFP fluorescence, allowing us to assess both increased and decreased differences. We assessed fluorescence by flow cytometry, which allowed us to quantify the average number of Cy3-DNA molecules per cell (Fig 2). Electroporation of equal moles of vectors resulted in the same number of DNA molecules per cell (Fig 2, squares). This finding demonstrates that vector delivery using electroporation is independent of vector length from 383 to 4,548 bp. Transfecting with an equal moles of vector means transfecting with more mass of DNA as vector length increases. Given our finding that cell entry is primarily dependent on the moles of DNA delivered, fewer vectors should be delivered per cell as vector length increases. We saw exactly this trend, except for transfection of the smallest vector, mv383, which resulted in fewer vectors per cell than the mv727 (Fig 2, circles).

**Fig 3:**
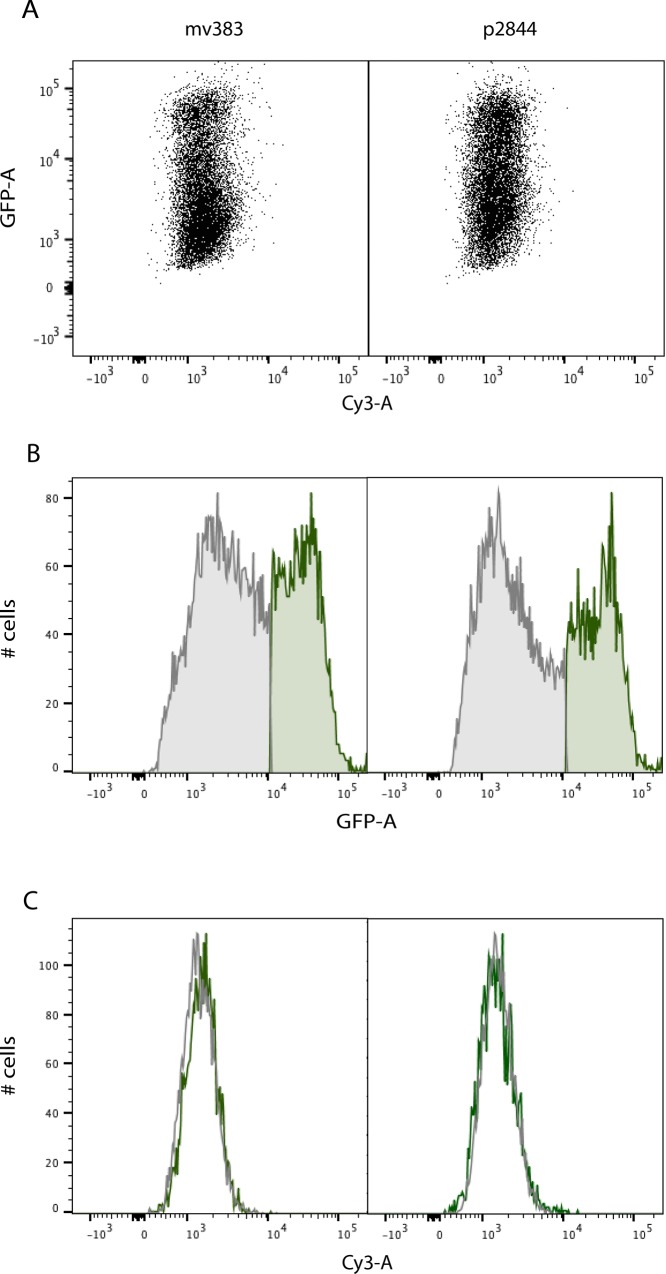
Lack of correlation between labeled DNA cell entry and GFP knockdown. HeLa-GFP cells were electroporated with Cy3-labeled DNA vectors. Representative results for the mv383 and p2844 vectors are shown. (A) GFP versus Cy3 fluorescence for cells transfected with 30 ng of mv383 or p2844 (B) Gating of GFP positive (green) and GFP negative cells (grey) (C) Cy3 fluorescence of GFP positive (green) versus GFP negative cells (grey) as gated in (B).

We expected to see a direct correlation between the number of molecules that enter a cell and vector-mediated GFP knockdown. Instead, we found no direct relationship between the number of Cy3-labeled vectors that entered cells and GFP knockdown (Fig 3A). In fact, when GFP-negative cells (shaded grey) and GFP-positive cells (shaded green) were gated separately (Fig 3B), Cy3 fluorescence in GFP-negative cells was virtually identical to Cy3 fluorescence in GFP-positive cells (Fig 3B and 3C). These results held true for all the vectors tested; two representative vectors (mv383 and p2844) are shown. These data show that whereas all vectors enter HeLa cells via electroporation, only a fraction of vectors that enter cells are subsequently expressed.

### Effect of DNA vector length on GFP knockdown

**Fig 4:**
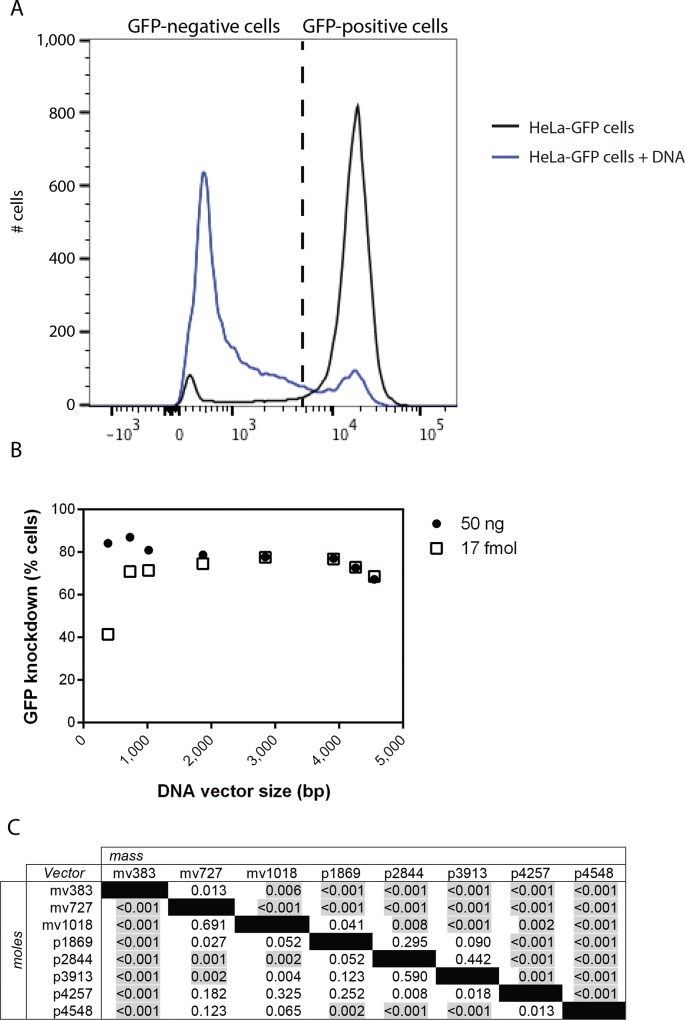
Flow cytometric analysis of the effect of DNA vector length on GFP knockdown. (A) Fluorescence of positive and negative controls showing the threshold between “GFP positive” and “GFP negative” cells. (B) 90,000 HeLa-GFP cells were transfected using electroporation with 50 ng of each of the eight DNA vectors and analyzed using flow cytometry (circles). In a separate experiment, 90,000 HeLa-GFP cells were transfected with 17 fmol of each of the eight DNA vectors (equivalent to 50 ng of the p4548) (squares). (C) *p*-values for a one-way ANOVA for pairwise comparisons of all the vectors are given, and statistically significant differences are highlighted.

To remove the likely possibility that the Cy3-LabelIT label interfered with knockdown, we separately used unlabeled vectors to analyze how altering vector length affected vector-mediated GFP knockdown. Untransfected HeLa-GFP cells were used to define the GFP-positive cell population (Fig 4A). The percentage of GFP-positive cells remaining after each electroporation was normalized to the percentage of GFP-positive cells of this control. We electroporated each vector into HeLa-GFP cells either with the same DNA mass (Fig 4B, circles) or with the same number of DNA molecules (Fig 4B, squares). Given our finding that equal moles of vectors were electroporated into cells in similar numbers (Fig 2), we expected that equal moles of each vector would cause identical GFP-knockdown. In contrast, GFP knockdown increased dramatically from vector length 383 bp to 727 bp, and continued to increase from 727 bp to 3,913 bp. Beyond 3,913 bp, however, GFP knockdown decreased with increasing lengths (Fig 4B, squares, Fig 4C, lower half). For knockdown of GFP, vectors of lengths 1,869, 2,844, 3,913, and 4,257 bp were equally the most efficient.

**Fig 5:**
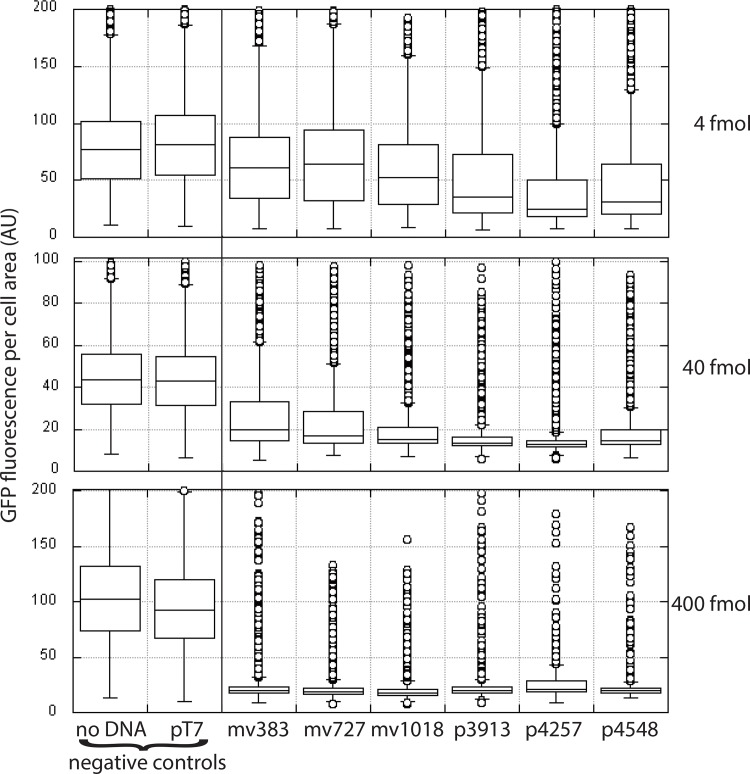
Fluorescence microscopy analysis of the effect of DNA vector length on GFP knockdown. 90,000 HeLa-GFP cells were transfected with the indicated concentrations of each vector, transferred after 24 hours to a 96-well glass bottom plate, incubated for another 24 hours, then fixed. Photographs of fields of cells from several wells from each transfection were taken. Images were analyzed using myImageAnalysis [21] to quantify GFP fluorescence and size for each cell, encompassing several thousand cells in each transfection. Data were plotted using a Tukey box plot where each outlier cell, defined by having a GFP per cell area more than 1.5 x interquartile range above the 75^th^ percentile, is denoted by a circle. Statistical differences were determined using a one-way ANOVA on ranks, *post hoc* Kruskal-Wallis test. In the top panel, all results except for those from mv383 and mv727 were statistically different from each other. In the middle panel, all but the two control samples were statistically significantly different from each other. In the bottom panel, all transfected cells were statistically significantly different from the controls, but only results for cells transfected with mv1018 differed from the other vectors.

To visualize directly the population of fluorescing cells, assess the effect of vector length on transfection by a separate method, and test multiple concentrations of vector doses, we used fluorescence microscopy and an automated method [21] to quantify GFP knockdown. We transfected each of the unlabeled vectors at 4, 40, or 400 fmol into HeLa-GFP cells. For each experiment, “no DNA added” and pT7 (a plasmid that encodes the same shRNA as the other vectors, but under a T7 promoter that does not express in mammalian cells), served as negative controls. From high resolution fluorescent images, cell area was calculated and GFP fluorescence per cell was quantified (S1 Fig) [21]. This process was done for the entirety of one visual field per well of a 96-well plate, with the total cells read ranging from 680 to 6,407 (depending on the number of cells visible in each field) for each transfection condition (Fig 5). When 4 or 40 fmol of each vector was transfected, GFP knockdown was strikingly similar to the results in Fig 4B, with decreasing GFP fluorescence with increasing vector length up to 4,257 bp. When 400 fmol of any vector were transfected, GFP for most cells was reduced to background levels.

One possible explanation for our finding that the longer vectors generally knocked down GFP more efficiently than the shorter vectors is that they have a greater mass per mole. When vectors were transfected with the same mass, there was a decrease in GFP-knockdown with increasing vector length above 727 bp (Fig 4B, circles, Fig 4C, upper half). This finding agrees with and expands upon previously published data that showed that an equal mass of 650 bp or 1,900 bp vectors transfect more efficiently than a 3,579 bp plasmid when electroporated [12]. These findings together suggest, then, that transfecting the same mass (more molecules) of the shortest vectors more than makes up for their reduced per molecule expression.

**Fig 6:**
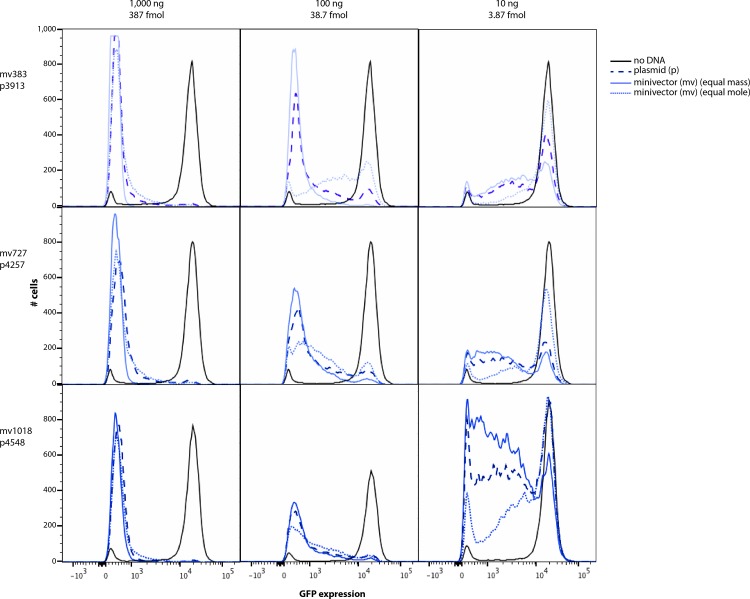
Direct comparison of transfection efficiency for three pairs of vectors. The larger vectors (dashed dark blue lines) were each transfected at one of three concentrations (mass and moles for these vectors are given) into 90,000 HeLa-GFP cells. For each concentration of the larger vector, each smaller vector was separately transfected into HeLa-GFP cells at two different concentrations. One concentration of the smaller vector was equivalent to an equal mass of minivector (solid light blue line), and the other concentration was equivalent to an equal number of molecules of smaller vector (dotted light blue line). Cells were analyzed using flow cytometry 48 hours post-transfection. Each panel is a representative result of an experiment that was performed at least twice in duplicate.

Because of the differences in GFP knockdown as a function of DNA length, and the opposing effects of moles and mass transfected, we designed an experiment to allow direct comparisons of mass and moles across multiple vector concentrations. We examined GFP knockdown comparing three different unlabeled plasmids (p3913, p4257, p4548) at three different concentrations to three different unlabeled minivectors (mv383, mv727, and mv1018) electroporated at either equal mass or equal mole concentrations for each plasmid concentration (Fig 6). Knockdown efficiency of the three minivectors was compared directly to the larger parent plasmid from which they were derived. Consistently, plasmids knocked down GFP more efficiently than their respective minivectors when transfected at equal moles (Fig 6). When transfected at equivalent masses, minivectors more efficiently knocked down GFP expression than their respective parent plasmids (Fig 6). These two results held true over a 100-fold change in vector concentration (Fig 6). These data show that increasing the concentration of the smaller vectors to an equal weight of the corresponding larger vector overcompensated for the decreased efficiency per mole. Thus, to yield an equal knockdown efficiency as a longer vector, a shorter vector must be transfected at a concentration higher than equal moles, but lower than equal mass. The role vector length plays in gene knockdown, therefore, is not simply a result of having a larger mass per molecule.

**Fig 7:**
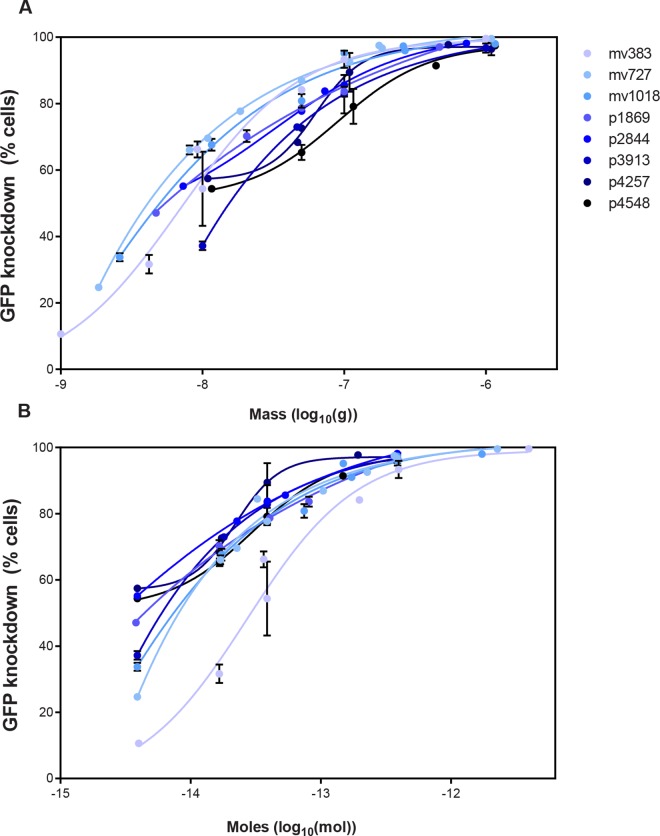
Effect of GFP knockdown as a function of DNA concentration. GFP knockdown was measured using a variety of DNA concentrations for each of the eight DNA vectors. Cells were analyzed using flow cytometry 48 hours post-transfection. The data were plotted either comparing the mass (A) or the number of molecules (B) of each of the different DNA vector lengths. Each panel shows the percentage of cells where GFP has been knocked down relative to the “no DNA” controls.

To investigate the effect of concentration on each vector and to determine whether the vectors had different concentration-knockdown relationships, we compared knockdown efficiency as a function of vector concentration for all eight vector lengths either with respect to mass transfected (Fig 7A) or with respect to moles transfected (Fig 7B) using unlabeled vectors. A two-way ANOVA showed that after mass was taken into account, vector length had a significant effect on GFP knockdown efficiency (*p* < 0.001). Separately, after moles were taken into account, vector length still had a significant effect on GFP knockdown efficiency (*p* < 0.001) (Table 1). Results from each vector were fit to a four-parameter logistics curve, similar to that done for a drug dose-response curve, which resulted in highly significant *r*^2^ values (from 0.9043 to 0.9977) (S1 Table). With respect to moles, mv383 had a drastically different dose-response curve from the other vectors, but even when mv383 was removed as a potential outlier, vector length still had a significant effect according to the two-way ANOVA (data not shown).

**Table 1:** Two-way ANOVA showing statistically significant effects of vector size. *P*-values of pairwise comparison of vectors after taking mass (top) and moles (left) into account.

		*mass*							
	*Vector*	mv383	mv727	mv1018	p1869	p2844	p3913	p4257	p4548
*moles*	mv383		0.998	0.212	0.006	0.010	<0.001*	0.003	<0.001*
	mv727	<0.001*		0.264	0.011	0.017	<0.001*	0.007	<0.001*
	mv1018	<0.001*	0.432		0.093	0.133	<0.001*	0.080	<0.001*
	p1869	<0.001*	0.090	0.303		0.873	0.111	0.939	0.004
	p2844	<0.001*	0.004	0.021	0.209		0.076	0.925	0.003
	p3913	<0.001*	0.177	0.533	0.666	0.086		0.065	0.107
	p4257	<0.001*	<0.001*	0.003*	0.094	0.840	0.024		0.001*
	p4548	<0.001*	0.125	0.415	0.796	0.121	0.854	0.039	

*Statistically significant at a 95% confidence level

The effective concentrations at which 50% or 75% of cells were transfected (EC_50_ and EC_75_) were calculated for each vector. Statistically significant differences were observed with vector size both as a function of mass and as a function of moles (S1 and S2 Tables). The biggest length differences in EC_50_ (mass concentrations) were seen between the smallest vector, mv383, and the larger vectors, p2844, p4257, and p4548 (S1 and S2 Tables). These differences in EC_50_ and EC_75_ values follow a similar trend as the results shown in Fig 4B, with knockdown efficiency per mole of vector electroporated increasing from 383 to 3,913 bp, then decreasing for vectors longer than 3,913 bp. All of the vectors tested have a similar general concentration-dependent knockdown relationship, and vector length affects transfection efficiency regardless of whether delivered at the same mass or the same moles.

## Discussion

This study is the first to focus on the effect of the variable of DNA vector length on electroporation-mediated transfection efficiency. Of our tested vectors, we saw maximal transfection efficiency per mass at 727 bp (Fig 4B, circles). We only worked at DNA concentrations where no obvious cell toxicity was seen in HeLa cells, which are easily transfected; many cell types are more difficult to transfect. Previously, however, we found that a 383 bp vector transfects Jurkat cells while its 3,913 bp parent vector, does not [7]. This previous finding suggests that cells less robust than HeLa may show exaggerated versions of the same trends we saw in HeLa cells.

We found that transgene expression in HeLa cells was modulated by DNA vectors in a dose-dependent manner, similar to the general dose-response observed with small molecule drugs. Although all the vector transfections followed a dose-response curve, vector length affected the EC_50_ and EC_75_ of each vector when mass or moles of vector transfected were taken into account. On the one hand, if mass were the sole determinant of knockdown efficiency, then we would have seen similar dose-response curves for different vectors when the results were plotted with respect to mass, which we did not (Fig 7A, S1 and S2 Tables, top halves). On the other hand, if number of molecules were the sole determinant of knockdown efficiency, then we would have seen similar dose-response curves among different vectors when the results were plotted with respect to number of molecules, which we also did not (Fig 7B, S1 and S2 Tables, bottom halves). Instead, we found that vector length affected the EC_50_ and the EC_75_ with respect to both the mass and the moles of DNA transfected. Therefore, when moles of vector is held constant, the improvements of gene knockdown seen with increasing DNA vector length, which are only seen up to 3,913 bp, cannot be accounted for solely by the resulting increased mass of vector. Therefore, vector length contributes to transfection efficiency in additional unknown ways.

Cell entry using cationic lipids is strongly dependent on the mass of DNA transfected because liposome formation is dependent on the number of negative charges on the DNA molecules, including those from filler DNA [11]. Electroporation is different. Instead of cell entry being primarily dependent on the mass of DNA transfected, cell entry by electroporation is most strongly correlated with the moles of DNA transfected (Fig 2). This is an important distinction when comparing vectors of different lengths and different transfection methods.

Because cell entry is not affected by vector length, but vector-mediated knockdown is, then some stage of transfection other than cell entry must be affected by vector length. DNA vectors transfected by electroporation can be transported to the nucleus along the cytoskeleton via endosomes [15,16]. Any vectors taking this route must then escape endosomes and be translocated to the nucleus; escape itself may be affected by vector length.

It is known that long DNA moves more slowly through the cytoplasm than short DNA [22]. Thus, another source of vector length dependence, for vectors that ended up in the cytoplasm following electroporation, would be the journey through the cytoplasm. Nuclear translocation occurs faster in actively dividing cells, such as HeLa, than in non-dividing cells because the nuclear membrane breaks down and reforms during cell division [23]. It does not seem likely that DNA vector length would affect accessing the nucleus through membrane breakdown because it would seem that as the membrane reforms, any vectors near the chromosomes would be engulfed in the new nucleus regardless of size.

Alternatively, vectors may enter the nucleus through nuclear pores. Whereas linear DNA up to 310 bp is capable of passively diffusing through nuclear pores, larger DNAs require active transport to reach the nucleus, which involves binding to translocation proteins [22]. It is well known that proteins are more likely to collide with longer DNAs than shorter DNAs [24], which means longer DNAs may be more likely to be bound by translocation proteins than shorter vectors. Our results indeed revealed increased GFP knockdown with increased vector length, but only up to 3,913 bp (Fig 4B, squares), suggesting that vector length may have an effect on nuclear pore translocation.

Even in the nucleus, vector length could affect transcription. Probabilistically, RNA polymerase is more likely to bind the longer the DNA is [24]. When a polymerase binds DNA, it slides along the DNA until it either finds the promoter region or releases the vector [25]. Thus, both three-dimensional and one-dimensional diffusion are important for transgene expression [24,25]. For longer vectors, even though the polymerase is more likely to bind the vector, one-dimensional sliding along the DNA is limited by its rate of release, meaning it will likely fall off the DNA after sliding for a certain number of bases [24]. Therefore, RNA polymerase may be less likely to reach the expression cassette on longer vectors, even though it is more likely to bind the vector initially. These protein-DNA dynamics suggest that the probability of gene expression increases as vector length increases until the vector is long enough that RNA polymerase is unlikely to reach the promoter by one-dimensional sliding, at which point, the frequency of promoter binding decreases as vector length increases. This logic tracks well with our results (Fig 4B, squares).

It is also possible that the shortest DNA vectors could be difficult to transcribe because there is less room to distribute the topological tension caused by the unwinding necessary for gene expression. It is important to keep in mind that mv383 is about half the molecular weight of the RNA polymerase II holoenzyme [26], which might explain why mv383-mediated GFP knockdown is less efficient than mv727.

The sum total of each step of transfection—cell entry, endosomal escape, nuclear localization, and transcription—determines the final efficiency of gene therapy delivery. While cell entry does not appear to be affected by vector length, transgene expression is affected by vector length.

## Materials and Methods

### Chemicals and reagents

Dulbecco’s modified eagle medium (DMEM), phosphate buffered saline, penicillin-streptomycin, and trypsin-ethylenediaminetetraacetic acid solutions were from Cellgro (Corning, Manassas, VA, USA). G418 was purchased from Sigma-Aldrich (St. Louis, MO, USA). Fetal bovine serum (FBS) was from Hyclone (ThermoFisher Scientific, Waltham, MA, USA). NucBlue fixed cell stain was from Invitrogen (Life Technologies, Carlsbad, CA, USA). All other chemicals were reagent grade or higher from VWR (Radnor, PA, USA).

### DNA vectors

Base pairs 1302–1639 and 1302–1930 from the plasmid pBR322 were inserted into the EcoRV site of p3913, the parent plasmid of mv383 [7] to create the parent plasmids for mv727 and mv1018, respectively. The 1,869 bp and 2,844 bp plasmids were created by cloning the 149 bp H1-GFPshRNA expression cassette into pDJC1 [8] and pUC18 (Novagen, Inc., Madison, WI, USA), respectively. The negative control pT7 is identical to p3913 except the H1 promoter was replaced with the viral T7 promoter. None of these vectors have any known nuclear targeting sequences, which are typically found in promoters and transcription factor binding sequences not present on these vectors [27]. Cloning the cassette into pDJC1 and pUC18 was done by Epoch (Sugarland, TX, USA). Minivectors were from Twister Biotech (Houston, TX, USA).

### Cell culture

HeLa cells were passaged in DMEM supplemented with 10% FBS and 100 μg/mL penicillin-streptomycin, and split at a ratio of 1:10 once every three to four days. As a quantitative reporter system of gene silencing, we used HeLa cells stably transfected with pd2EGFP-N1 (BD Sciences Clontech, Franklin Lakes, NJ, USA), which encodes a destabilized version of enhanced green fluorescent protein (eGFP) with a half-life of only two hours (most GFP variants have a half-life of 26 hours [20]). After transfection, cells were grown in the presence of the antibiotic G418 at a concentration of 800 μg/mL to maintain selection of pd2EGFP-N1. After a week of growth, to purify eGFP-expressing cells, cells were sorted using Fluorescence Activated Cell Sorting on a BD AriaI cell sorter (BD Sciences). The HeLa cells that were not expressing eGFP were discarded, and the cells expressing eGFP (HeLa-GFP) were grown in DMEM supplemented with 10% FBS and incubated at 37°C with 5% CO_2_.

### DNA labeling

DNA vectors were labeled using the Cy3-LabelIT reagent kit (Mirus, Madison, WI, USA). In each 50 μL reaction, 5 μg DNA was labeled with 1 μL of reagent. After one hour incubation at 37°C, unreacted reagent was removed using QiaQuick Nucleotide Removal kit (Qiagen). A NanoDrop ND-1000 Spectrophotometer (ThermoFisher Scientific) was used to measure absorbance at 260 nm and 550 nm, which was used to calculate the DNA and Cy3 concentrations, respectively.

### Cell transfection

The various vectors were transfected via electroporation with a Neon Transfection System (Invitrogen) using the manufacturer’s protocol. Briefly, cells were trypsinized, washed with PBS, and resuspended in “R buffer” from the Neon kit to a concentration of 1 x 10^7^ cells/mL. For each reaction, 9 μL of cells were mixed with 1 μL of DNA at the indicated concentrations and drawn into the 10 μL tip provided in the kit. Immediately after electroporation, cell were placed in 1 mL DMEM without antibiotics in a 12-well plate and incubated at 37°C.

### Flow cytometry

48 hours after electroporation, cells were collected, counted using a hemocytometer, and analyzed using flow cytometry. Experiments in which only GFP fluorescence was measured were performed with a BD LSRII flow cytometer. Experiments in which Cy3 and GFP fluorescence were both measured were performed using a BD LSRFortessa flow cytometer. Data were collected using FACSDiva software and analyzed using FlowJo version 10. For each experiment that analyzed Cy3 fluorescence, a standard curve was established using the median fluorescent intensity from Quantum™ R-PE Molecules of Equivalent Soluble Fluorescence (MESF) beads (Bangs Laboratories, Fishers, IN, USA). The number of molecules of R-PE on the standard curve was converted to the equivalent number of Cy3 fluorophores using the quantum yields of R-PE and Cy3 (0.82 and 0.15, respectively), which established the number of Cy3 fluorophores for any given Cy3 intensity.

### Fluorescence microscopy

HeLa-GFP cells were electroporated as above except that after 24 hours cells were collected, counted, and transferred to wells of a 96-well plate at a concentration of 8 000 cells/well. The cells were then fixed and permeabilized with 4% paraformaldehyde (Electron Microscopy Sciences, Hatfield, PA, USA), and stained with 4’,6-diamidino-2-phenylindole (DAPI). The images were collected on an epifluorescent IC200 image cytometer (Vala Sciences, San Diego, CA, USA) equipped with a scientific grade 16-bit sCMOS camera (Andor, Belfast, UK) and a Nikon 20X/0.75NA Plan Apo objective. Images were saved as uncompressed 16-bit grayscale TIF images, with each channel being saved as a separate image. Images were uploaded to and analyzed using a Pipeline Pilot (Biovia, San Diego, CA, USA) server running the myImageAnalysis web application [21]. The software defined and calculated the area of each cell in an image and the total GFP fluorescence in each cell.

### Statistical analyses

Statistical analyses for experimental significance were conducted using SigmaPlot. For each experiment with all eight DNA vectors at a single concentration, comparisons among vectors were made by one-way analysis of variance (ANOVA) and Holm-Sidak *post hoc* test with 5% trimming. For the fluorescence microscopy data, we performed a one-way ANOVA on ranks, *post hoc* Kruskal-Wallis test. This *post hoc* test uses Dunn’s method, which accounts for the uneven sample sizes. For experiments where the vectors were transfected at multiple concentrations, comparisons among vectors were made by two-way ANOVA with vector length and DNA concentration as independent variables.

To define maximal effective concentration, dose response curves were generated with four parameter log-logistic models, stratified by DNA length. From the data shown in Fig 7, EC_75_ values were generated from the fit curves and EC_50_ values were generated either directly from or extrapolated from the curves. In the tables with *p*-values, the shaded values indicate statistical significance at a 95% confidence level.

## Supporting Information

S1 Fig Representative fluorescent microscopy images. HeLa-GFP cells without (top) and with (bottom) DNA transfected. Images are shown with no filter (left) and with GFP and DAPI filters (right).
(PDF)

S1 Table EC_50_ and EC_75_ values for all eight vectors calculated from the best fit curves in Fig 7. (DOCX)

S2 Table One-way ANOVA of EC_50_ values showing statistically significant differences in best-fit curves for Fig 7A and 7B. (DOCX)

## References

[1] Wirth T, Parker N, Ylä-Herttuala S. History of gene therapy. Gene. 2013;525: 162–169. 10.1016/j.gene.2013.03.137 23618815

[2] Rao RC, Zacks DN. Cell and gene therapy. Dev Ophthalmol. 2014;53: 167–177. 10.1159/000357376 24732770 PMC4229945

[3] Wen Q., O'Reilly P., Dunne P.D., Lawler M., Van Schaeybroeck S., Salto-Tellez M. et al Connectivity mapping using a combined gene signature from multiple colorectal cancer datasets identified candidate drugs including existing chemotherapies. BMC Syst. Biol. 9 Suppl 5, S4 (2015). 10.1186/1752-0509-9-S5-S4 PMC4565135 26356760

[4] Kohn DB, Sadelain M, Glorioso JC. Occurrence of leukaemia following gene therapy of X-linked SCID. Nat Rev Cancer. 2003;3: 477–488. 10.1038/nrc1122 12835668

[5] Williams JA. Improving DNA vaccine performance through vector design. Curr Gene Ther. 2014;14: 170–189. 25142448 10.2174/156652321403140819122538

[6] Carnes A. E., Luke J.M., Vincent J.M, Anderson S., Schukar A., Hodgson C.P., et al Critical design criteria for minimal antibiotic-free plasmid vectors necessary to combine robust RNA Pol II and Pol III-mediated eukaryotic expression with high bacterial production yields. J. Gene Med. 12, 818–831 (2010). 10.1002/jgm.1499 20806425 PMC2959117

[7] Zhao N, Fogg JM, Zechiedrich L, Zu Y. Transfection of shRNA-encoding Minivector DNA of a few hundred base pairs to regulate gene expression in lymphoma cells. Gene Ther. 2011;18: 220–224. 10.1038/gt.2010.123 20962872 PMC3154479

[8] Catanese DJ, Fogg JM, Schrock DE, Gilbert BE, Zechiedrich L. Supercoiled Minivector DNA resists shear forces associated with gene therapy delivery. Gene Ther. 2012;19: 94–100. 10.1038/gt.2011.77 21633394 PMC3252587

[9] Chen Z-Y, He C-Y, Ehrhardt A, Kay MA. Minicircle DNA vectors devoid of bacterial DNA result in persistent and high-level transgene expression in vivo. Mol Ther J Am Soc Gene Ther. 2003;8: 495–500. 10.1016/s1525-0016(03)00168-0 12946323

[10] Gracey Maniar L. E., Maniar J.M., Chen Z., Lu J., Fire A.Z. & Kay M.A. Minicircle DNA vectors achieve sustained expression reflected by active chromatin and transcriptional level. Mol. Ther. J. Am. Soc. Gene Ther. 21, 131–138 (2013). 10.1038/mt.2012.244 PMC3538319 23183534

[11] Kreiss P., Cameron B., Rangara R., Mailhe P., Aguerre-Charriol O., Airiau M., et al Plasmid DNA size does not affect the physicochemical properties of lipoplexes but modulates gene transfer efficiency. Nucleic Acids Res. 27, 3792–3798 (1999). 10481017 10.1093/nar/27.19.3792 PMC148641

[12] Stenler S., Wilklander O.P.B., Badal-Tejedor M., Turunen J., Nordin J.Z., Hallengard D., et al Micro-minicircle gene therapy: Implications of size on fermentation, complexation, shearing resistance, and expression. Mol. Ther. Acids 2, e140 (2014). 10.1038/mtna.2013.67 PMC3910003 24399204

[13] Kobelt D. Schleef M., Schmeer M., Aumann J., Schlag P.M. & Walther W. Performance of high quality minicircle DNA for in vitro and in vivo gene transfer. Mol. Biotechnol. 53, 80–89 (2013). 10.1007/s12033-012-9535-6 22467123

[14] Martin T, Wysocki B, Wysocki T, Pannier A. Identifying intracellular pDNA losses from a model of nonviral gene delivery. IEEE Trans Nanobioscience. 2015; 10.1109/TNB.2015.2392777 25622323

[15] Rosazza C, Deschout H, Buntz A, Braeckmans K, Rols M-P, Zumbusch A. Endocytosis and Endosomal Trafficking of DNA After Gene Electrotransfer In Vitro. Mol Ther Nucleic Acids. 2016;5: e286 10.1038/mtna.2015.59 26859199 PMC4884790

[16] Rosazza C, Buntz A, Rieß T, Wöll D, Zumbusch A, Rols M-P. Intracellular tracking of single-plasmid DNA particles after delivery by electroporation. Mol Ther J Am Soc Gene Ther. 2013;21: 2217–2226. 10.1038/mt.2013.182 PMC3863794 23941812

[17] Layek B, Lipp L, Singh J. Cell penetrating peptide conjugated chitosan for enhanced delivery of nucleic acid. Int J Mol Sci. 2015;16: 28912–28930. 10.3390/ijms161226142 26690119 PMC4691089

[18] Safinya CR, Ewert KK, Majzoub RN, Leal C. Cationic liposome–nucleic acid complexes for gene delivery and gene silencing. New J Chem. 2014;38: 5164–5172. 10.1039/C4NJ01314J 25587216 PMC4288823

[19] Kim JA, Cho K, Shin MS, Lee WG, Jung N, Chung C, et al A novel electroporation method using a capillary and wire-type electrode. Biosens Bioelectron. 2008;23: 1353–1360. 10.1016/j.bios.2007.12.009 18242073

[20] Corish P, Tyler-Smith C. Attenuation of green fluorescent protein half-life in mammalian cells. Protein Eng. 1999;12: 1035–1040. 10611396 10.1093/protein/12.12.1035

[21] Szafran AT, Mancini MA. The myImageAnalysis project: a web-based application for high-content screening. Assay Drug Dev Technol. 2014;12: 87–99. 10.1089/adt.2013.532 24547743 PMC3934667

[22] Ludtke JJ, Zhang G, Sebestyen MG, Wolff JA. A nuclear localization signal can enhance both the nuclear transport and expression of 1 kb DNA. J Cell Sci. 1999;112: 2033–2041. 10341220 10.1242/jcs.112.12.2033

[23] Akita H, Kurihara D, Schmeer M, Schleef M, Harashima H. Effect of the compaction and the size of DNA on the nuclear transfer efficiency after microinjection in synchronized cells. Pharmaceutics. 2015;7: 64–73. 10.3390/pharmaceutics7020064 26066769 PMC4491651

[24] Gowers DM, Wilson GG, Halford SE. Measurement of the contributions of 1D and 3D pathways to the translocation of a protein along DNA. Proc Natl Acad Sci U S A. 2005;102: 15883–15888. 10.1073/pnas.0505378102 16243975 PMC1262116

[25] Halford SE, Marko JF. How do site-specific DNA-binding proteins find their targets? Nucleic Acids Res. 2004;32: 3040–3052. 10.1093/nar/gkh624 15178741 PMC434431

[26] Myer VE, Young RA. RNA polymerase II holoenzymes and subcomplexes. J Biol Chem. 1998;273: 27757–27760. 9774381 10.1074/jbc.273.43.27757

[27] Dean D, Strong D, Zimmer W. Nuclear entry of nonviral vectors. Gene Ther. 2005;12: 881–890. 10.1038/sj.gt.3302534 15908994 PMC4403635

