# Supplementary figures and images for: Effects of Circular DNA Length on Transfection Efficiency by Electroporation into HeLa Cells
Source: PLoS One. 2016 Dec 5;11(12):e0167537. doi: 10.1371/journal.pone.0167537 (PMC5137892; doi:10.1371/journal.pone.0167537)

No filter

GFP + DAPI

HeLa GFP cells  
(no DNA)

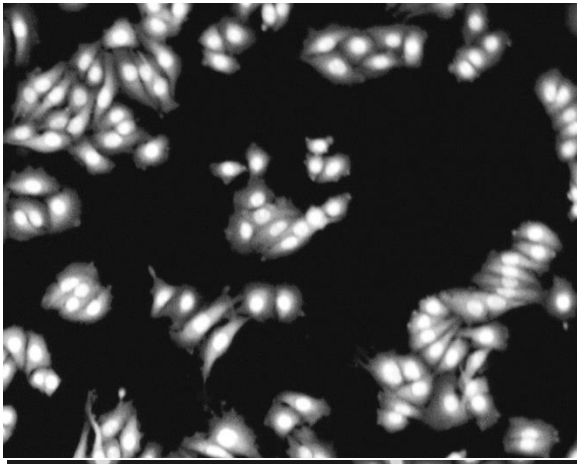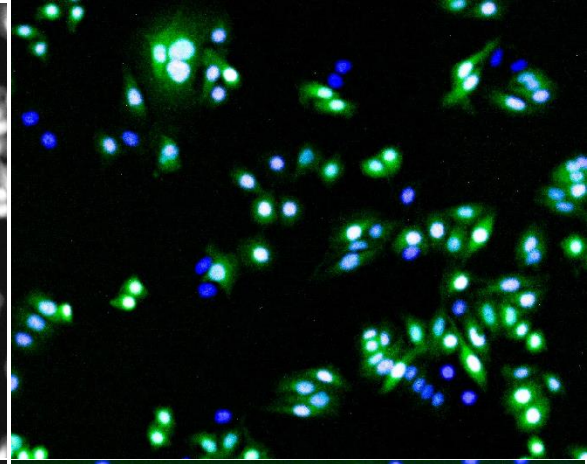

HeLa GFP cells  
(+1 000 ng  
p3913)

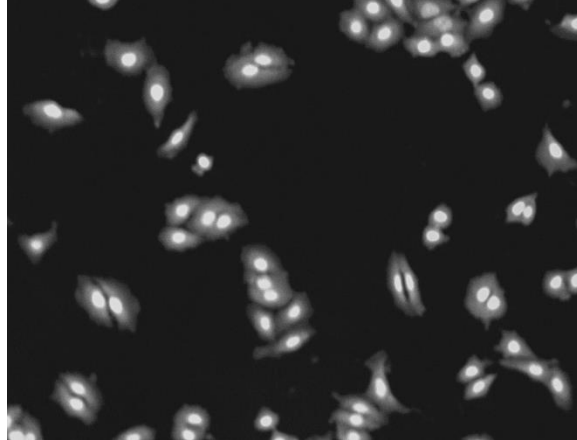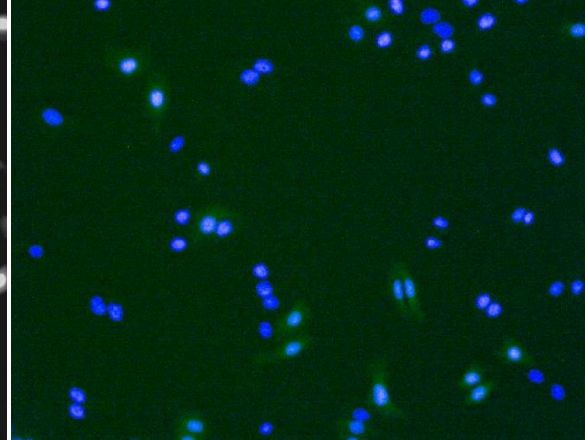

**Supplemental Figure 1**

Supplement: S1 Fig — HeLa-GFP cells without (top) and with (bottom) DNA transfected. Images are shown with no filter (left) and with GFP and DAPI filters (right). (PDF) [file pone.0167537.s001.pdf]
