# Supplementary material for: Effects of Circular DNA Length on Transfection Efficiency by Electroporation into HeLa Cells
Source: PLoS One. 2016 Dec 5;11(12):e0167537. doi: 10.1371/journal.pone.0167537 (PMC5137892; doi:10.1371/journal.pone.0167537)
Supplement: S1 Table — (DOCX) [file pone.0167537.s002.docx]

**S1 Table**

|  | *Vector* | *EC_50_* | *EC_75_* | *r^2^* |
| --- | --- | --- | --- | --- |
| *Mass* | mv383 | 8.08E-08 | 2.42E-07 | 0.9795 |
|  | mv727 | 8.06E-08 | 2.42E-07 | 0.9919 |
|  | mv1018 | 7.98E-08 | 2.40E-07 | 0.9842 |
|  | p1869 | 7.60E-08 | 2.28E-07 | 0.9938 |
|  | p2844 | 7.32E-08 | 2.20E-07 | 0.9977 |
|  | p3913 | 7.44E-08 | 2.23E-07 | 0.9043 |
|  | p4257 | 7.23E-08 | 2.17E-07 | 0.9666 |
|  | p4548 | 7.10E-08 | 2.13E-07 | 0.9749 |
| *Moles* | mv383 | 7.48E-15 | 6.92E-14 | 0.9795 |
|  | mv727 | 7.74E-15 | 2.22E-14 | 0.9919 |
|  | mv1018 | 7.81E-15 | 2.03E-14 | 0.9842 |
|  | p1869 | 7.69E-15 | 2.40E-14 | 0.9938 |
|  | p2844 | 7.59E-15 | 1.69E-14 | 0.9977 |
|  | p3913 | 7.86E-15 | 1.94E-14 | 0.9043 |
|  | p4257 | 7.68E-15 | 1.19E-14 | 0.9666 |
|  | p4548 | 7.41E-15 | 2.38E-14 | 0.9749 |
