# Supplementary material for: Effects of Circular DNA Length on Transfection Efficiency by Electroporation into HeLa Cells
Source: PLoS One. 2016 Dec 5;11(12):e0167537. doi: 10.1371/journal.pone.0167537 (PMC5137892; doi:10.1371/journal.pone.0167537)
Supplement: S2 Table — (DOCX) [file pone.0167537.s003.docx]

**Supplemental Table 2**

|  |  | *mass* | | | | | | | |
| --- | --- | --- | --- | --- | --- | --- | --- | --- | --- |
|  | *Vector* | mv383 | mv727 | mv1018 | p1869 | p2844 | p3913 | p4257 | p4548 |
| *moles* | mv383 |  | 0.272 | 0.378 | 0.329 | <0.001 | 0.255 | <0.001 | <0.001 |
|  | mv727 | <0.001 |  | 0.822 | 0.964 | <0.001 | 0.959 | <0.001 | <0.001 |
|  | mv1018 | <0.001 | 0.790 |  | 0.813 | <0.001 | 0.784 | <0.001 | <0.001 |
|  | p1869 | <0.001 | 0.916 | 0.739 |  | <0.001 | 1.000 | <0.001 | <0.001 |
|  | p2844 | 0.192 | 0.022 | 0.036 | 0.033 |  | <0.001 | 0.027 | <0.001 |
|  | p3913 | <0.001 | 0.903 | 0.699 | 1.000 | 0.017 |  | <0.001 | <0.001 |
|  | p4257 | 0.272 | 0.003 | 0.006 | 0.008 | 0.749 | 0.003 |  | 0.002 |
|  | p4548 | 0.009 | 0.175 | 0.260 | 0.201 | 0.312 | 0.146 | 0.142 |  |
